# Oral Factors as Predictors of Frailty in Community-Dwelling Older People: A Prospective Cohort Study

**DOI:** 10.3390/ijerph19031145

**Published:** 2022-01-20

**Authors:** Noriko Takeuchi, Nanami Sawada, Daisuke Ekuni, Manabu Morita

**Affiliations:** 1 Department of Preventive Dentistry, Okayama University Hospital, Okayama 700-8558, Japan; 2 Department of Preventive Dentistry, Graduate School of Medicine, Dentistry and Pharmaceutical Sciences, Okayama University, Okayama 700-8558, Japan; de422027@s.okayama-u.ac.jp (N.S.); dekuni7@md.okayama-u.ac.jp (D.E.); mmorita@md.okayama-u.ac.jp (M.M.)

**Keywords:** frailty, oral diadochokinesis, prospective cohort study

## Abstract

The purpose of this prospective cohort study was to identify predictors for frailty among possible oral factors in community-dwelling older people. Ninety-seven participants (≥60 years old) without frailty at baseline were included and assigned to either the robust or the frailty group after 2-year follow-up. The frailty was defined using the Japan Cardiovascular Health Study index. The numbers of present and functional teeth and periodontal disease severity were recorded. Bacterial counts on the dorsum of the tongue, oral moisture, tongue pressure, occlusal force, masticatory ability, and the oral diadochokinesis (ODK) rate were measured. Swallowing function, along with psychosocial status, relationships with communities and people, nutritional status, medical history, and comorbidities were evaluated using a questionnaire. The newly identified frailty group at follow-up showed significantly lower values in the number of teeth present, ODK/ta/sound and ODK/ka/sound rates, and clinical attachment level at baseline compared to the robust group. A logistic regression model showed a significantly negative association between the ODK/ta/sound rate at baseline and the incidence of frailty. Articulatory oral motor skill was found to be a predictor of frailty after two years.

## 1. Introduction

Frailty is an age-related medical syndrome characterized by a decline in strength, endurance, and physiological function that increases the likelihood of needing care or dying [1]. Frailty has been shown to be associated with negative health outcomes, such as hospitalization, institutionalization/dependency, and premature death [2]. Various factors associated with frailty are demographic and socioeconomic factors [3], systemic diseases [3,4], psycho-behavioral factors [3,5], and nutritional status [6].

Oral factors, such as oral health status and oral function, are also associated with frailty [7]. Castrejón-Pérez et al. [8] reported the association of decreased number of teeth present and severe periodontitis with development of frailty over a 3-year period in community-dwelling older people. Velázquez-Olmedo et al. [9] reported the association between poor oral health (caries, periodontal disease, dry mouth, etc.) and the incidence of frailty over a 12-month period.

On the other hand, there have also been studies investigating the association between oral function and frailty [8,9,10,11,12]. Most of these studies were cross-sectional, with few longitudinal studies [11,12,13]. Moreover, despite there being a variety of means to measure different kinds of oral function, some longitudinal studies focused on limited aspects of oral function, such as occluding pairs of teeth [11] and occlusal force [12]. Therefore, there are few reports that assessed a variety of oral functions simultaneously [12,13]. Watanabe et al. [13] reported that the risk of frailty was associated with lower bite force, masseter muscle thickness, and the oral diadochokinesis (ODK) rate.

We hypothesized that, of the various oral health status and oral functions, there may be factors that specifically predict frailty. The novelty of this longitudinal study was to evaluate various oral functions and oral health status simultaneously and to determine factors associated with the incidence of frailty. The purpose of this prospective cohort study was to identify oral factors that predict frailty in community-dwelling older people.

## 2. Materials and Methods

### 2.1. Study Participants

To assess the factors that predict frailty, a prospective cohort study was conducted. Participants who visited the Preventive Dentistry Clinic at Okayama University Hospital between November 2017 and January 2021 and gave consent were included in the study. The inclusion criteria were as follows: (1) aged 60 years or older; (2) able to understand the questionnaire; (3) able to walk independently; (4) regular visits to the preventive dental clinic; and (5) judged robust on frailty assessment. Exclusion criteria were data loss and no follow-up visits. All participants gave their written, informed consent to participate in this study. This study followed the STROBE guidelines.

### 2.2. Measurements

Well-trained dentists assessed frailty, oral status, and oral function. Prior to the study, for calibration purposes, the staff received instructions from the authors on the correct protocol for each measurement.

### 2.3. Assessment of Frailty

Frailty was defined using the Japan Cardiovascular Health Study (J-CHS) index based on the CHS index criteria [14]. The components of frailty in the J-CHS index are the same as in the original CHS index: weight loss, exhaustion, low level of activity, slowness, and weakness.

For weight loss, participants were considered to have experienced weight loss if they answered “yes” to the question, “Have you lost at least 2 kg in the past 6 months?” For exhaustion, participants were judged to be fatigued if they answered “yes” to the question, “In the past two weeks, have you ever felt tired for no reason?” Low level of activity was judged as positive if the participant answered “no” to both the questions, “Do you engage in moderate levels of physical exercise or sports aimed at health?” and “Do you engage in low levels of physical exercise aimed at health?” Slowness was defined as a slower walking speed under normal conditions. Participants were asked to walk at a normal walking speed along an 11-m straight walkway set on a flat floor. Walking time was measured at a distance of 5 m between marks located 3.0 m and 8.0 m from the starting point of the walk, and the average walking speed (m/s) was calculated. The cutoff value for slowness was set to be less than 1.0 m/s. Weakness was defined as a decrease in muscle strength based on grip strength, measured using a hand-held dynamometer (Takei Scientific Instruments Co., Ltd., Niigata, Japan). The sex-specific cutoff value for muscle weakness was defined as less than 26 kg for males and less than 18 kg for females.

In the J-CHS index, the absence of any of five factors (weight loss, exhaustion, low level of activity, slowness, and weakness) was classified as non-frailty, one or two factors as pre-frailty, and three or more factors as frailty. In this study, the participants were classified into two groups: a non-frailty group (robust group) and a pre-frailty and frailty group (frailty group).

### 2.4. Assessment of Oral Function

The evaluation of oral function was made with reference to the 2016 diagnostic criteria of the Japanese Geriatrics Society [15]. The examination methods for oral hypofunction were as follows.

#### 2.4.1. Bacterial Counts on the Dorsum of the Tongue

Samples for bacterial counts on the dorsum of the tongue were collected by rubbing the central part of the tongue with a sterile cotton swab [16]. A 1-cm distance was rubbed back and forth three times. The swab was set in a constant-pressure sample collection device, which was an accessory of a bacterial counter (Panasonic Healthcare, Tokyo, Japan). The number of bacteria in 1 mL of the sample was then measured using a bacterial counter. A device that maintains a constant rubbing pressure was used to reduce inter-operator variability [17]. Measurements were taken twice, and the average value was calculated.

#### 2.4.2. Oral Moisture

Using an oral moisture checker (Mucus^®^, Life Co., Ltd., Saitama, Japan), the degree of moisture of the buccal mucosa on the right and left sides and the mucosa at the center of the dorsum of the tongue, 1 cm away from the tip of the tongue, were measured [16]. With the special sensor cover provided, the sensor was pressed with a force of about 200 g to make uniform contact with the test surface and held for about 2 s until the measurement values were displayed. Measurements were made twice each, and the average value of all measurements was used as the oral moisture.

Oral moisture meters are suitable for quantitatively assessing the moisture content of the measurement site [18]. In addition, the moisture content of soft tissues in the oral cavity correlates with saliva secretion, and valid results have been reported for sensitivity and specificity in screening people with reduced saliva secretion [19]. It is also effective for comprehensively assessing oral moisture in participants who have difficulty producing saliva and cannot be actively tested for secretion.

#### 2.4.3. Tongue Pressure

Tongue pressure was measured using a tongue depressor (JMS Tongue Depressor TPM-01, JMS Corporation, Hiroshima, Japan) to obtain the maximum tongue pressure [16]. The maximum tongue pressure measured with the tongue depressor was the pressure generated when the balloon of the tongue depressor probe was crushed arbitrarily between the tongue and palate at the front of the palate and kept pressed with maximum force for several seconds. Measurements were taken with the dentures, if any, in use. Because of the difficulty in positioning the balloon, practice was performed before the measurement. Measurements were taken twice, with a break to avoid fatigue, and the average value was calculated.

#### 2.4.4. Occlusal Force

The occlusal forces were measured using an occlusal diagnostic system called the Dental Prescale System [16]. A pressure-sensitive film (Dental Prescale^®^ II, Fujifilm Corporation, Kuala Lumpur, Malaysia) and a pre-calibrated scanner (GT-X830, Seiko Epson Corp., Suwa, Japan) were used. Before the measurements, the participants sat in a dental chair in a natural posture, looking as far forward as possible so that the Frankfurt plane was parallel to the floor. Before measuring the occlusal force, the participants used the practice sheet several times. All participants were asked to bite the film for 3 s at maximum biting force. Denture wearers were measured with their dentures in place. The measurements were performed once.

#### 2.4.5. ODK

ODK was assessed to comprehensively measure the speed and elaboration of movements of the tongue and lips [16]. The pronunciations of /pa/, which reflects the motor function of the lips, /ta/, which reflects the motor function of the anterior tongue, and /ka/, which reflects the motor function of the posterior tongue, were evaluated. Participants were instructed to repeat each as fast as possible for 5 s, and the number of times they pronounced them was recorded by “Kenko-kun^®^ Handy” (Takei Scientific Instruments Co., Ltd., Niigata, Japan), and the number of times per second was calculated. The pronunciation of each sound was measured twice and the average value of each was calculated.

#### 2.4.6. Masticatory Ability

Masticatory ability was evaluated by the glucose concentration after chewing gummy jelly. Participants chewed a gummy jelly containing glucose (Glucolumn^®^, GC, Tokyo, Japan) as usual for 20 s [16]. They then gargled with 10 mL of water in their mouths and drained it into a filtration kit. The glucose concentration in the filtrate was measured with a glucose sensor (Glucosensor^®^ GS-II, GC, Tokyo, Japan). The measurement was carried out twice and the average value was calculated.

#### 2.4.7. Swallowing Function

The Japanese version of the Eating Assessment Tool (EAT-10) (Nestlé Nutrition Institute, Vevey, Switzerland) was used to assess swallowing function [16]. EAT-10 was a useful dysphagia screening tool. It consists of 10 questions about swallowing, each of which is answered on a 5-point scale (0: no problem, 4: severe problem). A lower total score indicates no problem with swallowing function, whereas a higher score indicates a severe problem.

### 2.5. Assessment of Oral Health Status

#### 2.5.1. Number of Present and Functional Teeth

The oral examination consisted of counting the number of teeth present and the number of functional teeth. The number of functional teeth was defined as natural teeth present and prostheses replacing missing teeth. Teeth with severe decay and retained roots were not considered present teeth [13]. The number of functional teeth was the number of current teeth plus the number of artificial teeth, such as pontics, implants, and dentures.

#### 2.5.2. Periodontal Status

Participants underwent a full-mouth periodontal examination at six sites per tooth (mesio-buccal, mid-buccal, disto-buccal, disto-lingual, mid-lingual, and mesio-lingual). The following parameters were evaluated and recorded: bleeding on probing (BOP), probing pocket depth (PPD), and clinical attachment level (CAL) [20]. These parameters were measured using a pocket probe (Williams Probe, Hu-Friedy Inc., Chicago, IL, USA) with standardized force ranging from 20 to 25 g.

### 2.6. Questionnaire Survey

#### 2.6.1. Subjective Oral Function

As a measure of subjective oral function, the difficulty of eating and swallowing was evaluated using a questionnaire [21]. “Do you have any difficulties eating tough foods?” and “Have you choked on your tea or soup recently?” were used to assess self-perceived oral function. If the respondents answered “yes” to each question, they were rated as those with difficulty in eating or swallowing.

#### 2.6.2. The World Health Organization—Five Well-Being Index (WHO-5)

The WHO-5 [22] is one of the most widely used scales for assessing psychological states to evaluate subjective psychological well-being. The WHO-5 has five positively worded items, and respondents rate each statement for the past two weeks. The scores range from 0 to 100, with higher scores indicating greater psychological well-being.

#### 2.6.3. Relationships with Communities and People

The Lubben social network scale 6-item version [23] assesses relationships with communities and people, and it consists of three questions that assess kinship ties and three similar questions that assess non-kinship ties. The items dealing with kinship include the following: “How many relatives do you see or keep in touch with at least once a month?”, “How many relatives do you see or talk to at least once a month?”, and “How many relatives do you feel free to talk to about your private matters?” These three items are repeated for connections other than relatives, replacing the word relatives with the word friends. The score for the entire scale is an equally weighted version of the six items, with a score range of 0–30.

#### 2.6.4. The Life-Space Assessment

The life-space assessment [24] measures mobility based on the distance through which a person reports moving during the 4 weeks preceding the assessment. Questions establish movement to specific life-space “levels”, ranging from within one’s dwelling to beyond one’s town. Frequency of movement and use of assistance (from equipment or persons) were also assessed. Specific levels were assessed by asking: “During the past 4 weeks, have you been to other rooms of your home besides the room where you sleep; to an area outside your home such as your porch, deck or patio, hallway of an apartment building, or garage; to places in your neighborhood, other than your own yard or apartment building; to places outside your neighborhood but within your town; and to places outside your town?” The overall scale score is based on dividing the living space into five levels and weighting them, with a score range of 0–1.

#### 2.6.5. Nutritional Status

Nutritional status was assessed with the Mini Nutritional Assessment (MNA)^®^ [25] (Nestlé Nutrition Institute, Vevey, Switzerland), BMI, and body weight. MNA^®^ is a 30-point scale and consists of 18 questions: anthropometric assessment (BMI, weight loss, upper middle arm circumference, calf circumference), global assessment (mobility, prescription medications, independent living, psychological stress or acute disability, pressure ulcers or bedsores, neuropsychological problems), dietary assessment (complete daily diet, decreased dietary intake, fluid intake, protein intake, fruit and vegetable intake, and feeding methods), and self-assessment (self-assessment of nutritional status and self-assessment of health status). The higher the total score, the better the nutritional status.

#### 2.6.6. Medical Interviews

A questionnaire was used to record medical history and number of medications taken. It requested information on the following conditions that have been linked to frailty: heart disease [26], osteoporosis [27], chronic obstructive pulmonary disease [28], knee osteoarthritis [29], anemia [30], depression [31], number of chronic diseases [32], Alzheimer’s disease [33], chronic kidney disease [34], rheumatoid arthritis [35], Parkinson’s disease [36], and stroke [36].

#### 2.6.7. Smoking and Alcohol History

Smoking and alcohol experience were confirmed by the questionnaire. Current and past experiences in smoking [12] and drinking [13] were categorized as having experience.

### 2.7. Statistical Analysis

All participants diagnosed as without frailty at baseline were included for statistical analysis, and they were encouraged to avoid dropping out of the study. Measurements were taken by skilled personnel to avoid measurement bias.

Participants of a cross-sectional study [20] were included in this study. Therefore, the sample size was not calculated.

Differences at baseline between the robust and frailty groups at reassessment were analyzed using the Mann–Whitney *U* test for continuous variables and the chi-squared test for categorical variables.

To examine the factors associated with the incidence of frailty during the study period, a binomial logistic regression analysis (variable reduction method) was performed with the two frailty-based groups (robust and frailty) as dependent variables. The independent variables were the items that showed *p* < 0.1 in the primary analysis.

The significance level was set at 0.05. Statistical analysis was performed using SPSS statistics version 26.0 (IBM Corp., Tokyo, Japan).

### 2.8. Ethical Considerations

This study was conducted in accordance with the tenets of the Declaration of Helsinki, and procedures involving human participants were approved by the ethics committee of Okayama University Graduate School of Medicine, Dentistry, and Pharmaceutical Sciences and Okayama University Hospital (approval number: 1803-038).

## 3. Results

At baseline, there were 107 participants who were robust on the frailty examination. Two years later, eight participants did not participate in the assessment at follow-up. Of the 99 study participants who participated in the follow-up assessment, 2 were excluded due to missing data. Thus, 97 participants (34 males and 63 females; follow-up rate: 90.6%) were included in the analysis.

Table 1 and Table 2 summarize the characteristics of all enrolled participants at baseline and at follow-up. At the time of reassessment, there were 63 participants in the robust group and 34 participants in the frailty group. In the two-group comparisons, the frailty group showed significantly lower values in the number of teeth present, ODK/ta/sound rate, ODK/ka/sound rate, and clinical attachment level at baseline (*p* = 0.062, 0.027, 0.032, and 0.042, respectively) compared to the robust group participants.

Table 3 shows the results from the logistic regression models for the association between oral status and onset of frailty. The ODK/ta/sound rate at baseline was significantly associated with onset of frailty (odds ratio = 1.85, 95% confidence interval = 1.02–3.35, *p* = 0.044).

## 4. Discussion

In this longitudinal cohort study, various oral functions were assessed and it was found that higher ODK/ta/sound rates were directly associated with lower frailty at 2 years. Although there have been several cross-sectional studies that have examined the relationships of oral function and oral health status with frailty, few longitudinal studies have been reported. Oral factors assessed in the longitudinal studies were current number of teeth and occlusal area [10], bite force of first molars [11], current number of teeth, periodontal status, subjective xerostomia and difficulty in chewing, and presence of cold water pain [37]. Some studies have examined the relationship between 16 oral functions and frailty [12]. Most of these studies assessed oral function as an individual function rather than with a comprehensive assessment. Therefore, oral health functions, including ODK, were simultaneously examined in the present study, and the ODK/ta/sound rate was found to be a predictor of frailty two years later.

The results of the present study show that ODK affects frailty, but the mechanism is not completely understood. One possibility is that poor ODK decreases nutritional status, which leads to frailty. Tanaka et al. [12] proposed an index combined with the current number of teeth, masticatory ability, articulatory oral motor ability (ODK), tongue pressure, and subjective eating and swallowing difficulties. The index was associated with physical frailty in their 2-year cohort study. They reported that oral frailty diagnosed using the index may affect physical frailty through nutritional status. A previous study [21] also reported that oral function, including ODK, was related to nutritional status two years later. It is possible that ODK affects nutritional status, and nutritional status affects frailty.

On the other hand, in the present cohort study, nutritional status was not associated with the onset of frailty at 2 years. In many articles reporting the relationship between nutrition and frailty, the percentage of subjects with good MNA scores ranged from 38.7% to 84.9% [38,39,40,41], but it was 91.8% in the present study. Since most of the participants had relatively good nutritional status, the effect of poor nutrition on frailty might have been small. In addition, nutritional status was not assessed between baseline and reassessment in this study. If nutritional status had been assessed between the baseline and 2-year follow-up, it might have been possible to clarify the relationships among oral function, frailty, and nutritional status.

Another possibility explaining the relationship between ODK and frailty might depend on sociological aspects. The presence of very unpleasant speaking problems was significantly associated with stress [42]. Communication disorders in older people, including voice and hearing loss, were important independent predictors of a decrease in the number of friends in social networks, certain elements of social support, and social participation [43]. The elaboration of movements of the tongue affects pronunciation. Poor tongue movement makes it difficult to speak and may be associated with stress-induced weakness. Of those over 70 years of age, the group with a reduced frequency of going out had higher subjective difficulty in mastication than the group with no reduction in the frequency of going out [44]. Poor tongue movement may have affected withdrawal.

Evaluation of ODK is relatively easy among the several measures to objectively evaluate oral function. The ODK measurement device is easy to use and highly reproducible. The number of times per second is displayed on the screen by simply pressing a button once and pronouncing a specific sound for 5 s. The measurement can be carried out by the participant himself/herself. The 3-month health program maintained improvements in the repetition rate of the monosyllables /pa/, /ta/, and /ka/ and physical function in older women [45]. Therefore, ODK evaluation appears useful to predict/prevent frailty.

Several longitudinal studies with follow-up periods of 2 to 5 years have reported that the number of teeth affects frailty [8,10,37]. They discussed the direct impact of the number of teeth on frailty, which may have been mediated by nutrition. In the present study, the number of teeth present did not affect frailty after 2 years. The median number of teeth present of the participants in the present study was 23.0, which was higher than that (19.0) reported by Iwasaki et al. [10]. Since most of the participants in the present study had regular visits to the university hospital and their oral condition was relatively well managed, it is possible that the effect on frailty was small.

Iwasaki et al. [11] observed that bite force was associated with frailty at 5 years. They considered that the effect of bite force on frailty may have been mediated by nutrition, since previous studies have reported that bite force affects nutritional status. In the present study, the value of bite force was higher in the robust group than in the frailty group, but the difference was not significant. Iwasaki et al. [11] measured the bite force at the first molar, but the total occlusal force of all the teeth was measured in the present study. The discrepancy between the study by Iwasaki et al. [11] and the present study might depend on the difference in measurement methods.

Castrejón-Pérez et al. [8] evaluated periodontal status using the modified periodontal screening and recording index [46] to determine the presence of severe periodontitis, and they reported its relationship with frailty after 3 years. Since the participants of this study were periodontal maintenance participants who had regular dental visits about every three months, their periodontal condition was considered to be relatively stable. Therefore, it is possible that the effect on frailty was low.

Frailty refers to a state of mental and physical fragility caused by the gradual decline of various mental and physical functions due to aging or illness. Frailty is not a condition that requires complete nursing care, and it is known that life functions can be improved to the previous state with appropriate lifestyle modification and treatment. Recently, frail oral function has been attracting attention as a condition that can be improved by appropriate intervention. In Japan, oral function improvement programs have been recommended as a countermeasure for oral function frailty. Although various exercises have been devised, most of them incorporate all aspects of oral function into the exercises indiscriminately without preliminary evaluation of individual oral factors. If ODK affects physical weakness among various oral functions, it would be beneficial to consider oral exercises that focus on tongue articulation.

There are several strengths of this research. The follow-up rate was high (90.6%). In addition, detailed oral data were acquired. The evaluation of frailty was objective. However, there were several limitations to this study. First, the participants were patients attending a university hospital and may have been in better physical condition than the general older population. External validity may not be high. In comparison with the study by Iwasaki et al. [10,11], in which unbiased participants were recruited, there is no significant difference with respect to the number of teeth in a similar age population. The external validity might not be so poor. Second, the socioeconomic factors reported to affect physical vulnerabilities [47] have not been examined in detail. Education [48], occupation [49], income [50], and wealth [50] were found to be associated with frailty in longitudinal studies.

## 5. Conclusions

Various oral functions were investigated in detail in older people (≥60 years old). Although oral health status, medical history, nutritional status, and psychosocial status were not associated with frailty, lower articulatory oral motor skill was found to be a predictor of frailty after two years.

## Figures and Tables

**Table 1:** Baseline characteristics (*n* = 97).

Variables	Mean ± SD/*n* (%)
**Sociodemographic data**	
Age	71.9 ± 5.4
Gender (men)	34 (35.1)
**Dental status**	
Number of teeth present (*n*)	20.8 ± 6.9
Number of functional teeth (*n*)	26.4 ± 3.6
**Periodontal status**	
%BOP	10.5 ± 8.9
PPD (mm)	2.1 ± 0.5
CAL (mm)	3.2 ± 1.2
**Oral function**	
Bacterial counts on the dorsum of the tongue (×10^6^) (cfu/mL)	18.6 ± 17.8
Oral moisture	28.6 ± 2.2
Tongue pressure (kPa)	32.8 ± 8.2
Occlusal force (*N*)	595.1 ± 421.9
ODK/pa/sounds (times/second)	6.0 ± 0.8
ODK/ta/sounds (times/second)	6.1 ± 0.7
ODK/ka/sounds (times/second)	5.8 ± 0.9
Masticatory ability (mg/dL)	177.3 ± 61.0
EAT-10 score	0.6 ± 2.0
**Subjective measures**	
Difficulty swallowing tea or soup (yes)	17 (17.5)
Difficulty in chewing foods as hard as squid or takuan (yes)	11 (11.3)
**Medical history**	
Heart disease	12 (12.4)
Osteoporosis	6 (6.2)
Chronic obstructive pulmonary disease	0 (0.0)
Knee osteoarthritis	0 (0.0)
Anemia	0 (0.0)
Stroke	1 (1.0)
Chronic kidney disease	2 (2.1)
Rheumatoid arthritis	1 (1.0)
Parkinson’s disease	1 (1.0)
Alzheimer’s disease	0 (0.0)
Number of medications	0.8 ± 0.8
**Nutritional status**	
MNA^®^	26.8 ± 2.4
BMI	22.7 ± 3.3
Body weight	57.6 ± 10.0
**Psychosocial status**	
WHO-5	72.0 ± 16.2
Lubben social network scale	16.8 ± 5.8
Life-space assessment	100 ± 18.2
**Smoking and alcohol history**	
Smoking experience	30 (30.9)
Drinking experience	45 (46.4)

SD: standard deviation, BOP: bleeding on probing, PPD: probing pocket depth, CAL: clinical attachment level, ODK: oral diadochokinesis, EAT-10: Eating Assessment Tool 10, MNA: mini nutritional assessment, BMI: body mass index, WHO-5: The World Health Organization—five well-being index.

**Table 2:** Baseline characteristics of the study population according to the presence of frailty at follow-up.

Variables	Robust Group (*n* = 63)	Frailty Group (*n* = 34)	*p*-Value
	Mean ± SD/*n*(%)	Mean ± SD/*n*(%)	
**Sociodemographic data**			
Age	72.1 ± 5.7	71.7 ± 5.0	0.483 *
Gender (men)	21 (33.3)	13 (38.2)	0.660 ^†^
**Dental status**			
Number of teeth present (*n*)	21.8 ± 6.7	19.1 ± 7.2	0.062
Number of functional teeth (*n*)	27.0 ± 2.0	25.3 ± 5.3	0.142
**Periodontal status**			
%BOP	11.4 ± 9.1	8.8 ± 8.2	0.141
PPD (mm)	2.2 ± 0.5	2.1 ± 0.5	0.258
CAL (mm)	3.1 ± 1.3	3.4 ± 1.0	0.042
**Oral function**			
Bacterial counts on the dorsum of the tongue (×10^6^) (cfu/mL)	19.3 ± 18.2	17.4 ± 17.2	0.631
Oral moisture	28.5 ± 2.2	28.7 ± 2.2	0.862
Tongue pressure (kPa)	32.8 ± 7.5	32.8 ± 9.6	0.717
Occlusal force (*N*)	642.4 ± 455.5	503.6 ± 335.5	0.225
ODK/pa/sounds (times/second)	6.2 ± 0.7	5.8 ± 0.8	0.124
ODK/ta/sounds (times/second)	6.3 ± 0.7	5.9 ± 0.7	0.027
ODK/ka/sounds (times/second)	5.9 ± 0.8	5.5 ± 0.9	0.032
Masticatory ability (mg/dL)	181.5 ± 62.4	169.4 ± 58.5	0.620
EAT-10 score	0.4 ± 1.1	1.1 ± 2.9	0.497
**Subjective measures**			
Difficulty swallowing tea or soup (yes)	9 (14.3)	8 (23.5)	0.274
Difficulty in chewing foods as hard as squid or takuan (yes)	6 (9.5)	5 (14.7)	0.509
**Medical history**			
Heart disease	8 (12.7)	4 (11.8)	1.000
Osteoporosis	4 (6.3)	2 (5.9)	1.000
Chronic obstructive pulmonary disease	0 (0.0)	0 (0.0)	-
Knee osteoarthritis	0 (0.0)	0 (0.0)	-
Anemia	0 (0.0)	0 (0.0)	-
Stroke	1 (1.6)	0 (0.0)	1.000
Chronic kidney disease	0 (0.0)	2 (5.9)	0.120
Rheumatoid arthritis	1 (1.6)	0 (0.0)	1.000
Parkinson’s disease	0 (0.0)	1 (2.9)	0.351
Alzheimer’s disease	0 (0.0)	0 (0.0)	-
Number of medications	0.7 ± 0.7	0.9 ± 0.8	0.246
**Nutritional status**			
MNA^®^	27.0 ± 2.0	26.5 ± 2.8	0.767
BMI	22.5 ± 3.5	23.2 ± 2.9	0.102
Body weight	56.8 ± 10.5	59.2 ± 8.8	0.132
**Psychosocial status**			
WHO-5	72.7 ± 16.5	70.7 ± 15.8	0.277
Lubben social network scale	17.2 ± 6.1	16.0 ± 5.2	0.294
Life-space assessment	100.4 ± 19	99.2 ± 17	0.646
**Smoking and alcohol history**			
Smoking experience	17 (27.0)	13 (38.2)	0.261
Drinking experience	30 (47.6)	15 (44.1)	0.832

* Mann–Whitney *U* test, ^†^ chi-square test, SD: standard deviation, BOP: bleeding on probing, PPD: probing pocket depth, CAL: clinical attachment level, ODK: oral diadochokinesis, EAT-10: Eating Assessment Tool 10, MNA: mini nutritional assessment, BMI: body mass index, WHO-5: The World Health Organization—five well-being index.

**Table 3:** Predictors of frailty on stepwise binary logistic regression analysis.

Model/Variables	OR	95% CI	*p*-Value
**Model1**			
ODK/ta/sound	1.847	1.017–3.354	0.044
ODK/ka/sound	―		
Number of teeth present	―		
CAL	―		
**Model2**			
ODK/ka/sound	1.597	0.977–2.608	0.062
Number of teeth present	―		
CAL	―		
**Model3**			
ODK/ta/sound	1.847	1.017–3.354	0.044
Number of teeth present	―		
CAL	―		

OR: odds ratio, CI: confidence interval, ODK: oral diadochokinesis, CAL: clinical attachment level.

## Data Availability

No new data were created or analyzed in this study. Data sharing is not applicable to this article.

## References

[1] Morley J.E., Vellas B., van Kan G.A., Anker S.D., Bauer J.M., Bernabei R., Cesari M., Chumlea W.C., Doehner W., Evans J. (2013). Frailty Consensus: A Call to Action. J. Am. Med. Dir. Assoc..

[2] Vermeiren S., Vella-Azzopardi R., Beckwée D., Habbig A.-K., Scafoglieri A., Jansen B., Bautmans I., Gerontopole Brussels Study Group (2016). Frailty and the Prediction of Negative Health Outcomes: A Meta-Analysis. J. Am. Med. Dir. Assoc..

[3] Fried L.P., Tangen C.M., Walston J., Newman A.B., Hirsch C., Gottdiener J., Seeman T., Tracy R., Kop W.J., Burke G. (2001). Frailty in Older Adults: Evidence for a Phenotype. J. Gerontol. A Biol. Sci. Med. Sci..

[4] Newman A.B., Gottdiener J.S., Mcburnie M.A., Hirsch C.H., Kop W.J., Tracy R., Walston J.D., Fried L.P. (2001). Cardiovascular Health Study Research Group Associations of Subclinical Cardiovascular Disease with Frailty. J. Gerontol. A Biol. Sci. Med. Sci..

[5] Avila-Funes J.A., Helmer C., Amieva H., Barberger-Gateau P., Le Goff M., Ritchie K., Portet F., Carrière I., Tavernier B., Gutiérrez-Robledo L.M. (2008). Frailty among Community-Dwelling Elderly People in France: The Three-City Study. J. Gerontol. A Biol. Sci. Med. Sci..

[6] Woods N.F., LaCroix A.Z., Gray S.L., Aragaki A., Cochrane B.B., Brunner R.L., Masaki K., Murray A., Newman A.B. (2005). Women’s Health Initiative Frailty: Emergence and Consequences in Women Aged 65 and Older in the Women’s Health Initiative Observational Study. J. Am. Geriatr. Soc..

[7] Hakeem F.F., Bernabé E., Sabbah W. (2019). Association between Oral Health and Frailty: A Systematic Review of Longitudinal Studies. Gerodontology.

[8] Castrejón-Pérez R.C., Jiménez-Corona A., Bernabé E., Villa-Romero A.R., Arrivé E., Dartigues J.-F., Gutiérrez-Robledo L.M., Borges-Yáñez S.A. (2017). Oral Disease and 3-Year Incidence of Frailty in Mexican Older Adults. J. Gerontol. A Biol. Sci. Med. Sci..

[9] Velázquez-Olmedo L.B., Borges-Yáñez S.A., Andrade Palos P., García-Peña C., Gutiérrez-Robledo L.M., Sánchez-García S. (2021). Oral Health Condition and Development of Frailty over a 12-Month Period in Community-Dwelling Older Adults. BMC Oral Health.

[10] Iwasaki M., Yoshihara A., Sato M., Minagawa K., Shimada M., Nishimuta M., Ansai T., Yoshitake Y., Miyazaki H. (2018). Dentition Status and Frailty in Community-Dwelling Older Adults: A 5-Year Prospective Cohort Study. Geriatr. Gerontol. Int..

[11] Iwasaki M., Yoshihara A., Sato N., Sato M., Minagawa K., Shimada M., Nishimuta M., Ansai T., Yoshitake Y., Ono T. (2018). A 5-Year Longitudinal Study of Association of Maximum Bite Force with Development of Frailty in Community-Dwelling Older Adults. J. Oral Rehabil..

[12] Tanaka T., Takahashi K., Hirano H., Kikutani T., Watanabe Y., Ohara Y., Furuya H., Tetsuo, Akishita M., Iijima K. (2018). Oral Frailty as a Risk Factor for Physical Frailty and Mortality in Community-Dwelling Elderly. J. Gerontol. A Biol. Sci. Med. Sci..

[13] Watanabe Y., Hirano H., Arai H., Morishita S., Ohara Y., Edahiro A., Murakami M., Shimada H., Kikutani T., Suzuki T. (2017). Relationship Between Frailty and Oral Function in Community-Dwelling Elderly Adults. J. Am. Geriatr. Soc..

[14] Tamaki K., Kusunoki H., Tsuji S., Wada Y., Nagai K., Itoh M., Sano K., Amano M., Maeda H., Hasegawa Y. (2018). The Relationship between Dietary Habits and Frailty in Rural Japanese Community-Dwelling Older Adults: Cross-Sectional Observation Study Using a Brief Self-Administered Dietary History Questionnaire. Nutrients.

[15] Minakuchi S., Kazuhiro T., Kazunori I., Takayuki U., Fumiyo T., Kan N., Junichi F., Kouichiro M., Ken Y., Manabu K. (2016). Deterioration of Oral Function in the Elderly-The Position Paperof Japanese Society of Gerodontology in 2016- (in Japanese). J. Gerodont..

[16] Takeuchi N., Sawada N., Ekuni D., Morita M. (2021). Oral Diadochokinesis Is Related to Decline in Swallowing Function among Community-Dwelling Japanese Elderly: A Cross-Sectional Study. Aging Clin. Exp. Res..

[17] Hisano A., Kikutani T., Tashiro H., Tamaru F., Hamada R. (2009). The Effect of Sampling Pressure Applied to the Tongue on Bacterial Counts. J. Gerodont..

[18] Fukushima Y., Kokabu S., Kanaya A., Hori N., Tateyama T., Sato T., Sakata Y., Kobayashi A., Araki R., Yanagisawa H. (2007). Experimental Examination of Appropriate Measurement Method of Oral Moisture Checking Device. J. Jpn. Oral Muco. Membr..

[19] Yamada H., Nakagawa Y., Nomura Y., Yamamoto K., Suzuki M., Watanabe N., Saito I., Seto K. (2005). Preliminary Results of Moisture Checker for Mucus in Diagnosing Dry Mouth. Oral Dis..

[20] Sawada N., Takeuchi N., Ekuni D., Morita M. (2021). Oral Function, Nutritional Status and Physical Status in Japanese Independent Older Adults. Gerodontology.

[21] Iwasaki M., Motokawa K., Watanabe Y., Shirobe M., Inagaki H., Edahiro A., Ohara Y., Hirano H., Shinkai S., Awata S. (2021). A Two-Year Longitudinal Study of the Association between Oral Frailty and Deteriorating Nutritional Status among Community-Dwelling Older Adults. Int J. Environ. Res. Public Health.

[22] Awata S., Bech P., Koizumi Y., Seki T., Kuriyama S., Hozawa A., Ohmori K., Nakaya N., Matsuoka H., Tsuji I. (2007). Validity and Utility of the Japanese Version of the WHO-Five Well-Being Index in the Context of Detecting Suicidal Ideation in Elderly Community Residents. Int. Psychogeriatr..

[23] Lubben J., Blozik E., Gillmann G., Iliffe S., von Renteln Kruse W., Beck J.C., Stuck A.E. (2006). Performance of an Abbreviated Version of the Lubben Social Network Scale among Three European Community-Dwelling Older Adult Populations. Gerontologist.

[24] Baker P.S., Bodner E.V., Allman R.M. (2003). Measuring Life-Space Mobility in Community-Dwelling Older Adults. J. Am. Geriatr. Soc..

[25] Vellas B., Villars H., Abellan G., Soto M.E., Rolland Y., Guigoz Y., Morley J.E., Chumlea W., Salva A., Rubenstein L.Z. (2006). Overview of the MNA--Its History and Challenges. J. Nutr. Health Aging.

[26] Liperoti R., Vetrano D.L., Palmer K., Targowski T., Cipriani M.C., Lo Monaco M.R., Giovannini S., Acampora N., Villani E.R., Bernabei R. (2021). Association between Frailty and Ischemic Heart Disease: A Systematic Review and Meta-Analysis. BMC Geriatr..

[27] Liu L.-K., Lee W.-J., Chen L.-Y., Hwang A.-C., Lin M.-H., Peng L.-N., Chen L.-K. (2015). Association between Frailty, Osteoporosis, Falls and Hip Fractures among Community-Dwelling People Aged 50 Years and Older in Taiwan: Results from I-Lan Longitudinal Aging Study. PLoS ONE.

[28] Marengoni A., Vetrano D.L., Manes-Gravina E., Bernabei R., Onder G., Palmer K. (2018). The Relationship between COPD and Frailty: A Systematic Review and Meta-Analysis of Observational Studies. Chest.

[29] Bindawas S.M., Vennu V., Stubbs B. (2018). Longitudinal Relationship Between Knee Pain Status and Incident Frailty: Data from the Osteoarthritis Initiative. Pain Med..

[30] Palmer K., Vetrano D.L., Marengoni A., Tummolo A.M., Villani E.R., Acampora N., Bernabei R., Onder G. (2018). The Relationship between Anaemia and Frailty: A Systematic Review and Meta-Analysis of Observational Studies. J. Nutr. Health Aging.

[31] Setiati S., Soejono C.H., Harimurti K., Dwimartutie N., Aryana I.G.P.S., Sunarti S., Budiningsih F., Mulyana R., Dwipa L., Sudarso A. (2021). Frailty and Its Associated Risk Factors: First Phase Analysis of Multicentre Indonesia Longitudinal Aging Study. Front. Med..

[32] de Breij S., van Hout H.P.J., de Bruin S.R., Schuster N.A., Deeg D.J.H., Huisman M., Hoogendijk E.O. (2021). Predictors of Frailty and Vitality in Older Adults Aged 75 Years and Over: Results from the Longitudinal Aging Study Amsterdam. Gerontology.

[33] Buchman A.S., Boyle P.A., Wilson R.S., Tang Y., Bennett D.A. (2007). Frailty Is Associated with Incident Alzheimer’s Disease and Cognitive Decline in the Elderly. Psychosom. Med..

[34] Shen Z., Ruan Q., Yu Z., Sun Z. (2017). Chronic Kidney Disease-Related Physical Frailty and Cognitive Impairment: A Systemic Review. Geriatr. Gerontol. Int..

[35] Andrews J.S., Trupin L., Yelin E.H., Hough C.L., Covinsky K.E., Katz P.P. (2017). Frailty and Reduced Physical Function Go Hand in Hand in Adults with Rheumatoid Arthritis: A US Observational Cohort Study. Clin. Rheumatol.

[36] Jazbar J., Locatelli I., Kos M. (2021). The Association between Medication or Alcohol Use and the Incidence of Frailty: A Retrospective Cohort Study. BMC Geriatr..

[37] Ramsay S.E., Papachristou E., Watt R.G., Tsakos G., Lennon L.T., Papacosta A.O., Moynihan P., Sayer A.A., Whincup P.H., Wannamethee S.G. (2018). Influence of Poor Oral Health on Physical Frailty: A Population-Based Cohort Study of Older British Men. J. Am. Geriatr. Soc..

[38] Bollwein J., Volkert D., Diekmann R., Kaiser M.J., Uter W., Vidal K., Sieber C.C., Bauer J.M. (2013). Nutritional Status According to the Mini Nutritional Assessment (MNA^®^) and Frailty in Community Dwelling Older Persons: A Close Relationship. J. Nutr. Health Aging.

[39] Boulos C., Salameh P., Barberger-Gateau P. (2016). Malnutrition and Frailty in Community Dwelling Older Adults Living in a Rural Setting. Clin. Nutr..

[40] Chang S.-F., Lin P.-L. (2016). Prefrailty in Community-Dwelling Older Adults Is Associated with Nutrition Status. J. Clin. Nurs..

[41] Jürschik P., Botigué T., Nuin C., Lavedán A. (2014). Association between Mini Nutritional Assessment and the Fried frailty index in older people living in the community. Med. Clin..

[42] Kim Y.S., Kim H.-N., Lee J.-H., Kim S.-Y., Jun E.-J., Kim J.-B. (2017). Association of Stress, Depression, and Suicidal Ideation with Subjective Oral Health Status and Oral Functions in Korean Adults Aged 35 Years or More. BMC Oral. Health.

[43] Palmer A.D., Carder P.C., White D.L., Saunders G., Woo H., Graville D.J., Newsom J.T. (2019). The Impact of Communication Impairments on the Social Relationships of Older Adults: Pathways to Psychological Well-Being. J. Speech Lang. Hear. Res..

[44] Mikami Y., Watanabe Y., Motokawa K., Shirobe M., Motohashi Y., Edahiro A., Nakajima J., Osuka Y., Inagaki H., Fujiwara Y. (2019). Association between Decrease in Frequency of Going out and Oral Function in Older Adults Living in Major Urban Areas. Geriatr. Gerontol. Int..

[45] Iwao-Kawamura Y., Shigeishi H., Uchida S., Kawano S., Maehara T., Sugiyama M., Ohta K. (2021). Changes in Physical and Oral Function after a Long-Term Care Prevention Program in Community-Dwelling Japanese Older Adults: A 12-Month Follow-Up Study. Healthcare.

[46] Landry R.G., Jean M. (2002). Periodontal Screening and Recording (PSR) Index: Precursors, Utility and Limitations in a Clinical Setting. Int. Dent. J..

[47] Wang J., Hulme C. (2021). Frailty and Socioeconomic Status: A Systematic Review. J. Public Health Res..

[48] Etman A., Kamphuis C.B.M., van der Cammen T.J.M., Burdorf A., van Lenthe F.J. (2015). Do Lifestyle, Health and Social Participation Mediate Educational Inequalities in Frailty Worsening?. Eur J. Public Health.

[49] Brunner E.J., Shipley M.J., Ahmadi-Abhari S., Valencia Hernandez C., Abell J.G., Singh-Manoux A., Kawachi I., Kivimaki M. (2018). Midlife Contributors to Socioeconomic Differences in Frailty during Later Life: A Prospective Cohort Study. Lancet Public Health.

[50] Arrighi Y., Rapp T., Sirven N. (2017). The Impact of Economic Conditions on the Disablement Process: A Markov Transition Approach Using SHARE Data. Health Policy.

